# Quality by Design for Optimizing a Novel Liposomal Jojoba Oil-Based Emulgel to Ameliorate the Anti-Inflammatory Effect of Brucine

**DOI:** 10.3390/gels7040219

**Published:** 2021-11-18

**Authors:** Marwa H. Abdallah, Heba S. Elsewedy, Amr S. AbuLila, Khaled Almansour, Rahamat Unissa, Hanaa A. Elghamry, Mahmoud S. Soliman

**Affiliations:** 1 Department of Pharmaceutics, College of Pharmacy, University of Ha’il, Ha’il 81442, Saudi Arabia; a.abulila@uoh.edu.sa (A.S.A.); kh.almansour@uoh.edu.sa (K.A.); ru.syed@liveuohedu.onmicrosoft.com (R.U.); m.soliman@uoh.edu.sa (M.S.S.); 2 Department of Pharmaceutics and Industrial Pharmacy, Faculty of Pharmacy, Zagazig University, Zagazig 44519, Egypt; hanaaelghamry@yahoo.com; 3 Department of Pharmaceutical Sciences, College of Clinical Pharmacy, King Faisal University, Alhofuf 31982, Saudi Arabia; helsewedy@kfu.edu.sa; 4 Department of Pharmaceutics and Industrial Pharmacy, Faculty of Pharmacy, Al-Azhar University, Cairo 11651, Egypt

**Keywords:** PEGylated liposomes, emulgel, anti-inflammatory, Brucine, jojoba oil, transdermal drug delivery

## Abstract

One of the recent advancements in research is the application of natural products in developing newly effective formulations that have few drawbacks and that boost therapeutic effects. The goal of the current exploration is to investigate the effect of jojoba oil in augmenting the anti-inflammatory effect of Brucine natural alkaloid. This is first development of a formulation that applies Brucine and jojoba oil int a PEGylated liposomal emulgel proposed for topical application. Initially, various PEGylated Brucine liposomal formulations were fabricated using a thin-film hydration method. (2^2^) Factorial design was assembled using two factors (egg Phosphatidylcholine and cholesterol concentrations) and three responses (particle size, encapsulation efficiency and *in vitro* release). The optimized formula was incorporated within jojoba oil emulgel. The PEGylated liposomal emulgel was inspected for its characteristics, *in vitro*, *ex vivo* and anti-inflammatory behaviors. Liposomal emulgel showed a pH of 6.63, a spreadability of 48.8 mm and a viscosity of 9310 cP. As much as 40.57% of Brucine was released after 6 h, and drug permeability exhibited a flux of 0.47 µg/cm^2^·h. Lastly, % of inflammation was lowered to 47.7, which was significant effect compared to other formulations. In conclusion, the anti-inflammatory influence of jojoba oil and Brucine was confirmed, supporting their integration into liposomal emulgel as a potential nanocarrier.

## 1. Introduction

Nanotechnology is a technique that is concerned with manipulating materials into new matters within a nano-range [1]. Predominantly, nanotechnology is applied in many fields, especially the medical field through drug delivery systems. Currently, most the active pharmaceutical ingredients suffer from low solubility and bioavailability, which could be avoided via applying nanotechnology in developing drug delivery systems. A number of nanocarriers have been successfully developed, including nanoparticles, nanoemulsion, ethosome, niosome and liposome [2].

Liposome is a nanocarrier that gained a great concern since it offers a variety of advantages over conventional systems and free drugs [3]. It represents a biodegradable phospholipid bilayer system with a spherical shape characterized by its high efficiency, ability to improve drug solubility and capability to incorporate both hydrophilic and hydrophobic drugs [4]. Currently, liposomes are applied in many aspects such as in vaccination, cancer therapy, anti-inflammatory [5] and bacterial and fungal infection [6]. It could be delivered via different routes of administration including oral, parenteral transdermal and topical delivery [7].

One obstacle that researchers might face after developing liposomes is related to their stability issues [8]. In order to avoid such hurdles, it is better to shield liposomes with certain hydrophilic polymers to form steric hindrance barrier that could improve their stability [9]. Poly-ethylene glycol (PEG) is a widely used polymer that is adopted for coating pharmaceutical formulations in order to enhance their stability and/or protect the encapsulated drug [10]. Interestingly, PEGylation was extensively applied for intravenous drug targeting; nevertheless, recent studies have revealed the impact of PEGylated liposome in topical applications [11,12].

Developing liposomes for topical drug delivery system could face certain difficulties related to their low viscosities and improper application to the skin [13]. Integrating liposomes into emulgel formulation could possibly increase the viscosity of the formulation and facilitate its application over the affected area [14]. Emulgel is a combination of emulsion and gel such that it gains the benefit of both systems. Topical application of emulgel has shown several advantages such as being thixotropic, straightforward spreadability and good patient compliance [15]. Emulgel has been investigated for incorporating different drugs to be applied for various purposes such as mefenamic acid for analgesic and anti-inflammatory activity [16], flaxseed extracts for wound healing [17] and Metronidazole for antifungal and antibacterial activity [15]. 

Right now, the use of many natural products for treating several diseases has been established since they are safe and exhibit substantial pharmacological behavior [18]. Brucine is a natural substance that is obtained from seeds of *Nux vomica* tree and being one of its essential alkaloids [19]. It has been reported to influence the cardiovascular system and has antitumor, antibacterial, analgesic and anti-inflammatory effects [20]. However, its low solubility constitutes a great hurdle to its efficacy, which necessitates the finding of new formulations that could overcome the drawbacks and boost its activity.

Another category of natural product is plant oils, which are regarded as a nourishing source of good health and characterized by their availability and cheapness [21]. One of these oils is jojoba oil, obtained from *Simmondsia chinensis* seeds [22]. It is yellow in color and comprises numerous components, mainly flavonoids, polyphenols and alkaloids. Recently, jojoba oil has been extensively used in skin care products [23]. In addition, it exhibited great effectiveness in handling skin disorders such as dermatitis, eczema, acne and psoriasis, besides its potential for promoting wound healing [24]. Moreover, it can be used as a therapy in treating cancer, kidney and liver dysfunction, and acute lung injury [25], and it has exhibited certain anti-inflammatory activities [26]. Jojoba oil can be used in combination with several nanocarriers to form emulsion, microemulsion [27], nanoemulsion and emulgel [25]. Assimilation of jojoba-oil-based emulgel with liposomal formulation of Brucine could support the topical delivery and effectiveness of the drug. 

Quality by design (QbD) is a systematic and organized technique for developing a unique product depending on pre-established factors and studying their influence on certain responses in order to attain the most optimized formula. It includes several tools, such as three-level full factorial, central composite design (CCD) and Box–Behnken design [28]. The most commonly used of them is Central Composite Design (CCD), which helps in selecting the optimized formula and in predicting the model relying on statistical analysis of variance (ANOVA) and definite equations [29].

In these contexts, our target was to develop PEGylated liposomal formulation containing Brucine that was subjected to optimization by implementing a 2^2^ full factorial design and discovering the impact of definite independent variables on the examined dependent variables. The optimized PEGylated Brucine-loaded liposome was combined with the developed jojoba oil based emulgel. Then, the PEGylated liposomal emulgel loaded with Brucine was analyzed for physical and chemical properties. Ultimately, the developed liposomal emulgel was examined for its skin permeation and its anti-inflammatory influence through an exploration of the initiative of incorporating jojoba oil with Brucine. 

## 2. Results and Discussion

### 2.1. Experimental Design

#### 2.1.1. Fitting the Model

Operating CCD software gave rise to generating a matrix of 11 examinations distributed as four factorial points, four axial and three central points. Table 1 displays the impact of every independent variable on the experiential dependent variables of diverse PEGylated liposome formulations.

#### 2.1.2. Analysis of the Design

In order to predict and recognize the model, statistical analysis of data should be performed. It was understood that the model’s *p*-values should be significant, and this is achieved if the value being less than 0.05. Additionally, lower F-values of the responses could result in more error in the model, and thus greater F-values are more desirable. Another vital parameter is the lack of fit, which is preferred to be non-significant in order to fit the data with the model [30].

It is clear in Table 2 that *p*-values of all the dependent variables R_1_, R_2_, and R_3_ remained smaller than 0.0001, which confirmed that the effect of the independent variables on the observed dependent ones was significant [31]. Moreover, the model’s F-values in R_1_, R_2_, and R_3_ seemed to be significant. Further, lack of fit in the three cases, R_1_, R_2_, and R_3_, exhibited non-significant values (*p* > 0.05) of 8.53, 4.39 and 1.75 and consistent *p*-values of 0.1086, 0.1971 and 0.4069 for R_1_, R_2_ and R_3_, respectively.

### 2.2. Characterization

#### 2.2.1. Influence of the Independent Variables on Particle Size (R_1_)

Particle size of the developed PEGylated Brucine-loaded liposomes is considered one of the essential independent variables that were estimated as apparent in Table 1. It was settled that particle size of all formulations ranged between 191 ± 2.9 nm and 287 ± 4.5 nm for F9 and F4, respectively. In the data, it was obvious that increasing both EPC and cholesterol concentration resulted in a remarkable increase the particle size of all the formulations. This could be attributed to the increased thickness of the liposomal layers as more cholesterol will be dispersed within the lipid bilayers [32]. This was in accordance with Wu et al., who noticed that papain liposomes showed a decrease in the particle size upon decreasing phospholipid concentration [33]. The following equation emphasized the previous findings, as it explains the positive influence of A and B independent variables on the detected R_1_ response, where the positive sign point to a synergistic action, while negative one defines antagonistic effect [34]:

R_1_ = 118.108 + 1.29882A + 1.6935B

**Figure 1:**
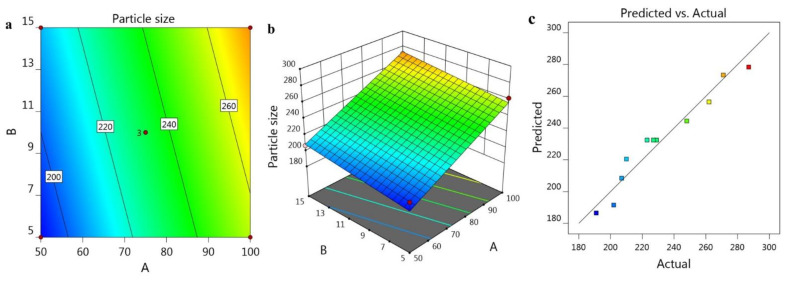
Representing (**a**) 2D-Contour plot, (**b**) 3D-Response Surface Plot, and (**c**) linear correlation plot between predicted against actual values.

Furthermore, Figure 1a,b shows 2D Contour and 3D-response surface plots that help in elucidating the influence of A and B independent variables on the R_1_ response. Figure 1c and facts in Table 2 clarified the linearity of the data through the interrelation between the adjusted R^2^ value (0.9347) and the predicted one (0.8948).

#### 2.2.2. Influence of the Independent Variables on EE (R_2_)

Encapsulation efficiency for all developed PEGylated liposomal preparations were implemented, and as apparent in Table 1, it ranged from 41.6 ± 2.1 to 71.6 ± 1.6% for F9 and F11, respectively. Referring to the data, it was noticed that percentage of encapsulation efficiency increased upon increasing EPC concentration, which proved a positive influence of variable A. This is probably ascribed to the larger particle size of the formed liposome upon increasing EPC concentration, which gives more space for the drug to be encapsulated. Our findings are in agreement with those of Tefas et al., who established that encapsulation of curcumin and doxorubicin significantly increased upon increasing phospholipid concentration [35]. On the other hand, cholesterol concentration exerted a negative influence on the % of EE, where decreasing cholesterol concentration with constant EPC concentration resulted in a remarkable increase in percentage EE. Interestingly, cholesterol and hydrophobic drugs favor staying in the hydrophobic area of the liposomal membrane, and thus competition between them could occur and lower encapsulation accordingly attained [36]. Wu et al. confirmed our results since the study revealed that using more cholesterol leads to lowering the stability and rigidity of the liposome [37]. Remarkably, it was revealed from the results that there is a correlation between liposomal particle size, and its capability to encapsulate drug since increasing particle size resulted in considerable increment in % of EE as declared in a previous study [38]. The mathematical equation obtained from the design proved our prospects as it clarifies the synergistic influence of A and antagonistic action of B independent variables on the comeback of R_2_.

R_2_ = 31.6342 + 0.400298A − 0.536556B

**Figure 2:**
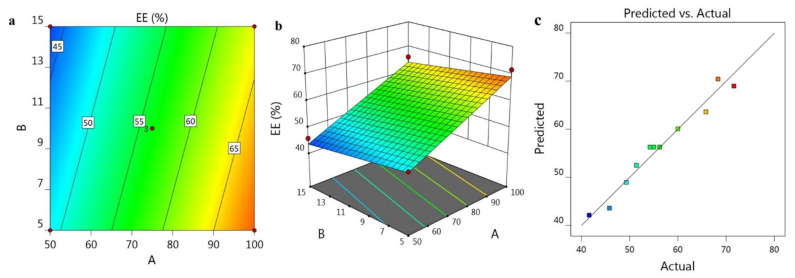
Representing (**a**) 2D-Contour plot, (**b**) 3D-Response Surface Plot and (**c**) linear correlation plot between predicted against actual values.

The impact of the independent variables A and B on R_2_ is graphically represented in Figure 2a,b exhibiting 2D Contour and 3D-response surface plot. Furthermore, Figure 2c supports the linearity of the data where the adjusted R^2^ value (0.9595) was in correlation with the predicted one (0.9329).

#### 2.2.3. Influence of the Independent Variables on *In Vitro* Release (R_3_)

**Figure 3:**
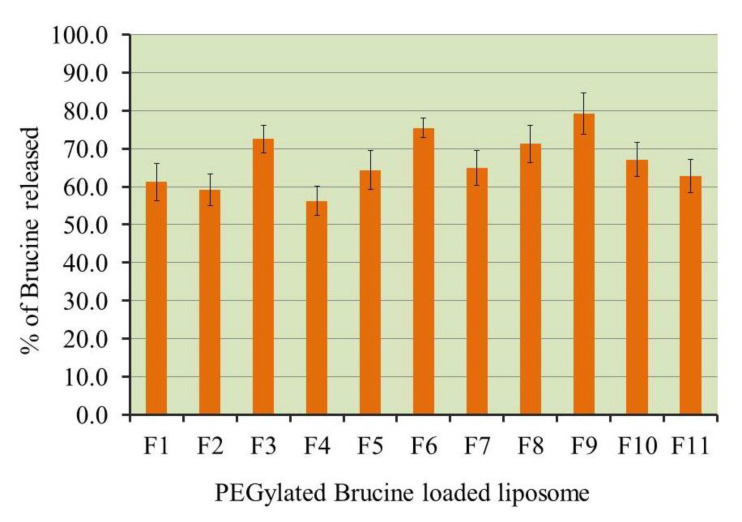
*In vitro* release of Brucine from different PEGylated liposome formulations in phosphate buffer pH 7.4 at 37 °C. Results are expressed as mean ± SD of three experiments.

The *in vitro* release characteristic of Brucine from all liposomal formulations under investigation was efficiently evaluated. The results were depicted in Figure 3 and Table 1. The study lasted for 6 h and the % of drug released ranged between 56.3 ± 3.9 and 79.2 ± 5.5%. It was obvious that increasing both EPC and cholesterol concentration exerted a negative antagonistic effect on the dependent variable R_3_. This could be due to the formerly revealed fact that large liposomes were obtained upon increasing concentration of A and B independent variables. Larger nanocarriers usually provide larger surface area and accordingly lower the percentage of Brucine released [39]. Another explanation could be accredited to the presence of DSPE-PEG in the liposomal formulation that offers more stability for the formulation in addition to the rigidization formed at the surface of the liposome by DSPE-PEG that led to a decrease in the % of drug *in vitro* release [40].

**Figure 4:**
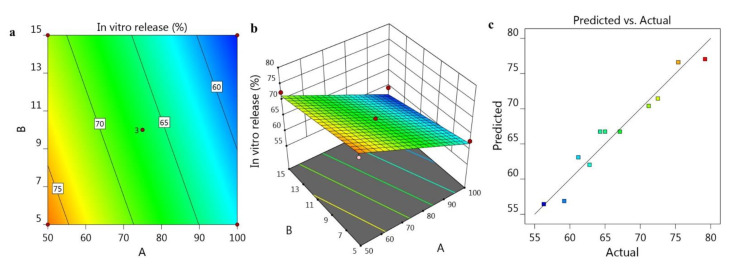
Representing (**a**) 2D-Contour plot, (**b**) 3D-Response Surface Plot and (**c**) linear correlation plot between predicted against actual values.

The former data obtained from *in vitro* release investigation are demonstrated in Figure 4a,b screening 2D-Contour and 3D-Response Surface Plots. Further, the linearity of the data was corroborated as presented in Figure 4c displaying the linear correlation plot where, the observed adjusted R^2^ value was 0.9797 and the predicted one was 0.9341. Both values of R^2^ are in reasonable agreement with each other as the variation between them was less than 0.2. In addition, the mathematical equation provided below would confirm the interrelation among the independent variables and their examined dependent one:

R_3_ = 93.763 − 0.291427A − 0.516053B

### 2.3. Optimizing the Developed PEGylated Liposomal Formulations Using CCD

**Figure 5:**
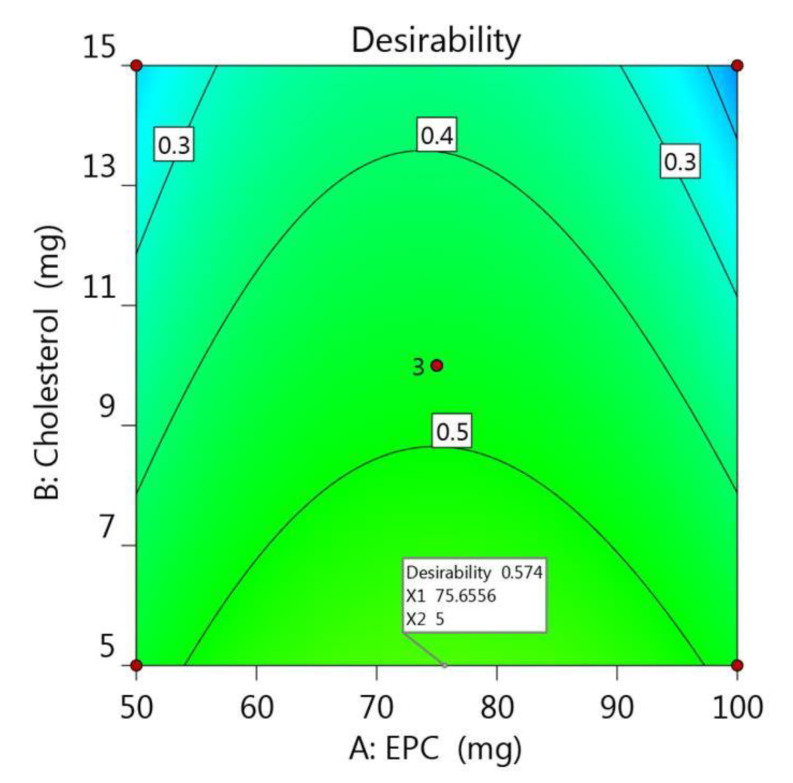
Desirability graph presenting the influence of independent variables A and B on the overall dependent variables R_1_, R_2_ and R_3_.

**Figure 6:**
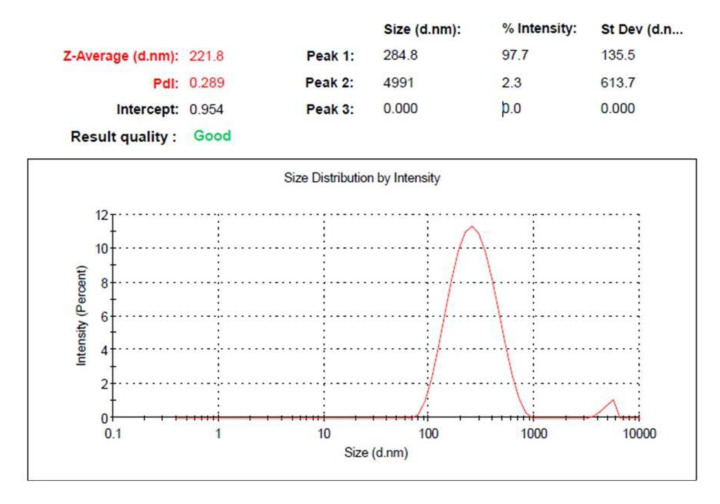
Particle size and PDI of optimized PEGylated Brucine liposomal formulation.

Following constructing the design using CCD, it is very important to specify the optimum formulation via point prediction method and relying on the greater desirability obtained to provide the best formulation with appropriate features [41]. In the numerical optimization, the independent and dependent variables were guided toward requisite goals that help in selecting ideal formula. In the current investigation, A and B were adjusted to be within range in addition to R_1_, while the criteria shifted toward maximizing both R_2_ and R_3_. As a consequence, A and B values were proposed to be 75.64 and 5 mg, respectively, in addition to the expected values for the optimized formulation that seemed to fulfill the optimization process as illustrated in Table 3 and the desirability value (0.574) as in Figure 5. The data of the optimal point suggested from the software were used to develop a new optimized formulation, and upon comparing its observed result, it was found to be very close to the expected one. The distribution curve of the optimized PEGylated Brucine liposomal formulation is exhibited in Figure 6, demonstrating the particle size (221.8 ± 2.04) and allied PDI (0.289 ± 0.62).

### 2.4. Stability Study of the Optimized PEGylated Brucine Liposomal Formulation

**Figure 7:**
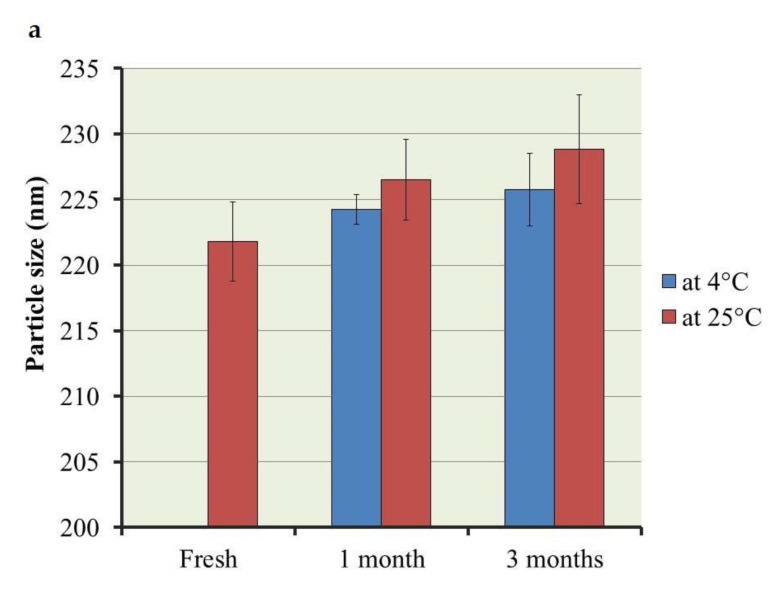

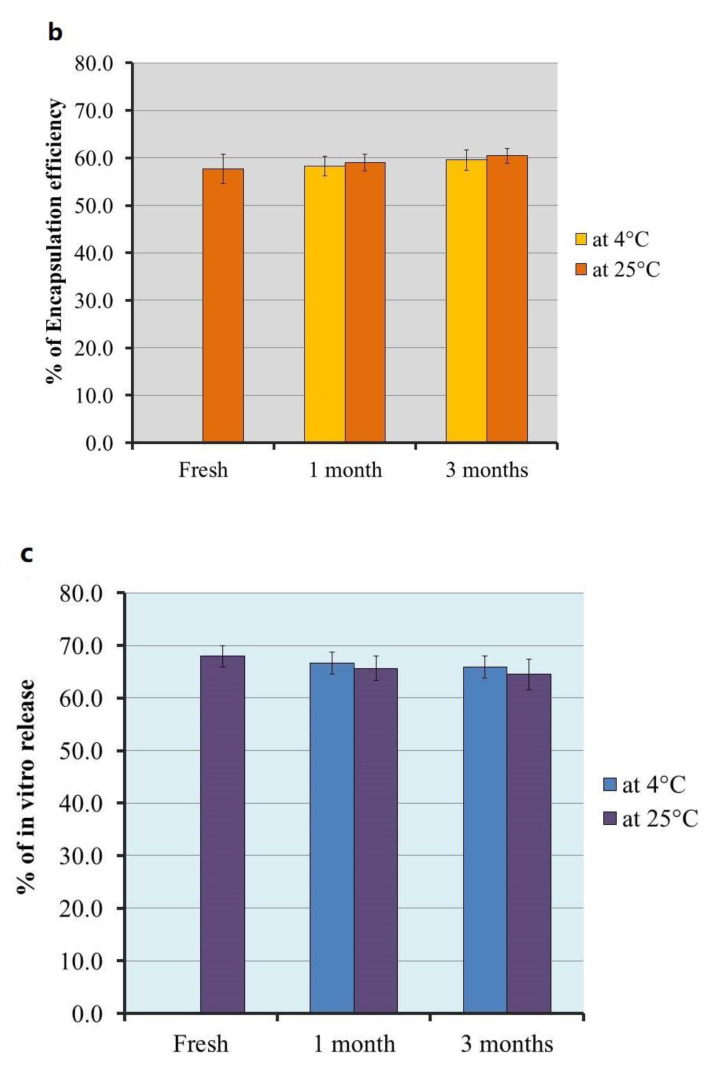
Outline of stability study for optimized PEGylated Brucine liposomal formulation for 1 and 3 months at 4 °C and 25 °C in terms of (**a**) particle size; (**b**) EE and (**c**) *in vitro* drug release, compared to freshly prepared formulation. Results are expressed as mean ± SD of three experiments.

Estimating stability of the optimized liposomal formulation was accomplished over 1 and 3 months following storage at 4 ± 1 °C and at 25 ± 1 °C, and results are discussed in Figure 7. The results revealed that non-significant differences (*p* < 0.05) were observed upon comparing fresh preparation with that following storage, which considered confirmed evidence for the formulation stability and warrants the efficiency of liposome as a nanocarrier. This stability could be attributed to the presence of DSPE-PEG since it provides a steric hindrance for the liposomal membrane [11]. 

As stated by the preceding attained results, the optimized Brucine liposomal formulation was assimilated with the pre-formulated jojoba oil-based emulgel via gentle stirring in order to develop a novel liposomal jojoba oil-based emulgel encapsulating Brucine, which was used in certain evaluations.

### 2.5. Estimating the Features of Developed PEGylated Liposomal Emulgel Encapsulating Brucine

The formulation characterization was demonstrated in terms of several aspects. Physical inspection revealed that the preparation appeared as a smooth, homogenous emulgel whose screening for physical appearance is convenient. With reference to pH value validation, it was recorded as 6.63 ± 0.25, which was compatible with pH of the skin and appropriate to preclude any skin irritation. Spreadability of the formulation was 48.8 ± 2.7 mm in addition to the viscosity, which was 9310 ± 336 cP, indicating adequate result for topical preparation to be easily applied over the skin.

### 2.6. In Vitro Drug Release from Liposomal Emulgel

**Figure 8:**
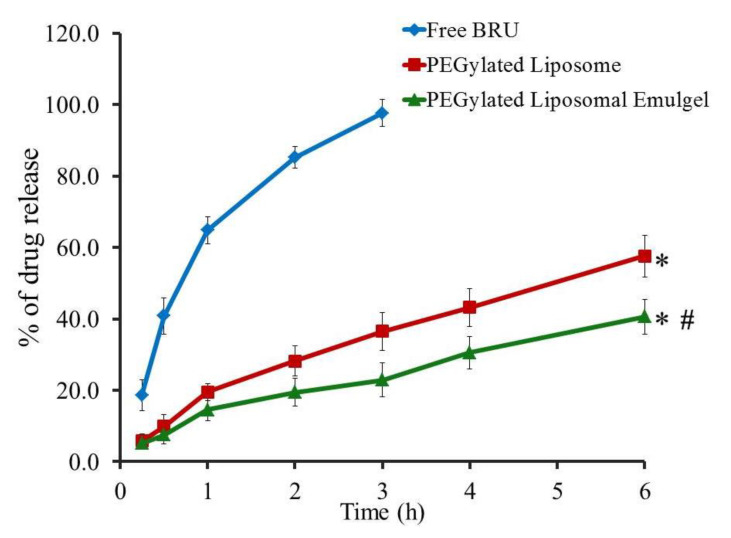
*In vitro* release outline of Brucine from free Brucine suspension, PEGylated liposome and PEGylated liposomal emulgel in phosphate buffer pH 7.4 at 37 °C. Results are identified with respect to the mean ± SD of three experiments. * *p* < 0.05 compared to free drug; # *p* < 0.05 compared to PEGylated liposome formulation.

The behavior of drug release from Brucine suspension, liposomal formulation and optimized liposomal emulgel fabricated with jojoba oil was successfully prolonged for 6 h in phosphate buffer pH 7.4, and the release profile is displayed in Figure 8. It is obvious that almost 97.6 ± 3.8% of Brucine was released from free drug suspension within almost 3 h. The *in vitro* release of Brucine from PEGylated liposomal formulation and optimized liposomal emulgel was 57.53 ± 5.85 and 40.57 ± 4.82%, respectively, which displayed significant lower profile compared to Brucine released from suspension (*p* < 0.05). Alternatively, the *in vitro* release of Brucine from PEGylated liposomal formulation is significantly higher than that released from optimized PEGylated liposomal emulgel (*p* < 0.05). This could be attributed to the presence of jojoba oil and gelling agent in the emulgel formulation, which provides greater viscosity of the preparation and consequently lowers the rate of encapsulated drug diffusion [42].

### 2.7. Permeation Studies

**Figure 9:**
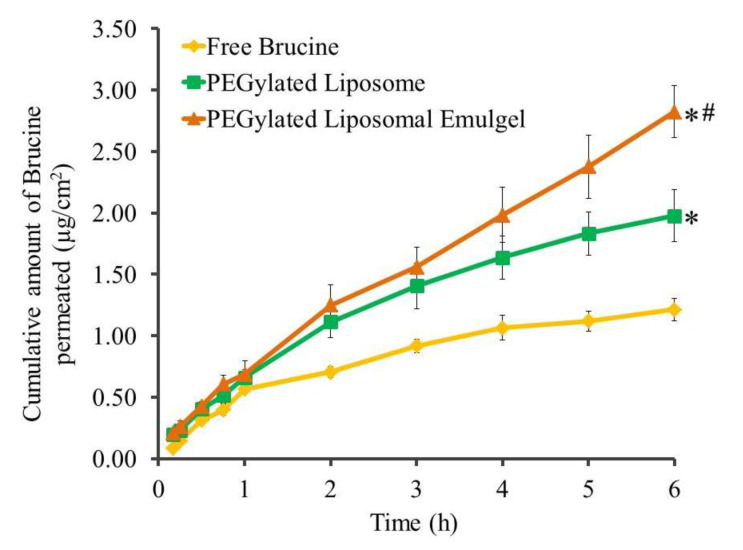
Outline of *ex vivo* permeability study of Brucine from diverse preparations through rat skin membrane. Results are identified in respect of mean ± SD (n = 3). * (*p* < 0.05) compared to free Brucine; # compared to PEGylated liposome formulation.

Permeation studies were executed for Brucine formulations over 6 h across rat skin membranes, and the results, expressed as the amount permeated, are presented in Figure 9. In addition, the permeability parameters represented by SSTF and ER values are displayed in Table 4. A statistically significantly lower amount of Brucine was permeated from free Brucine suspension (0.202 ± 0.015 µg/cm^2^·h) (*p* < 0.05), which is comparable with other formulations under examination. On the other hand, the SSTF of Brucine from PEGylated liposomal emulgel was 0.47 ± 0.035 µg/cm^2^·h, enhancing the permeability by 2.33 ± 0.174 folds, which was significantly higher than the flux from PEGylated liposome (0.321 ± 0.028 µg/cm^2^·h) with ER value 1.603 ± 0.142 (*p* < 0.05). In fact, higher flux from PEGylated formulations could be attributed to the integration of DSPE-PEG, which would bind to water molecules and consequently increase the hydration of stratum corneum, resulting in augmented skin permeability [8]. Higher flux from liposomal emulgel than liposomal formulation itself could be related to the colloidal characteristics of surfactant as well as the presence of jojoba oil, where both work as a permeation enhancer that improved the permeability [43,44]. In addition, surfactant has the capability to interact with the lipids of rat skin, increasing its fluidity, which could seemingly improve the drug permeation. Another explanation for higher permeation detected in liposomal emulgel could be ascribed to the dual action of surfactant and emulsion that forms the emulgel preparation, which could probably diffuse across the narrow pores of the membrane [45]. Our results are in agreement with a study done by Shehata et al. that proved significant higher permeability of insulin from niosomal emulgel when compared to insulin solution and niosomal gel [46].

### 2.8. In Vivo Study

#### 2.8.1. *In Vivo* Skin Irritation Test

Careful examination of animal back skin treated with investigated formulations was performed for checking any sensitivity reactions that might occur. No inflammation, irritation, erythema or edema was recognized on the inspected area during the whole 7 days of the investigation, which reflected safety of the formulations.

#### 2.8.2. *In Vivo* Anti-Inflammatory Study: Carrageenan-Induced Rat Hind Paw Edema Method

**Figure 10:**
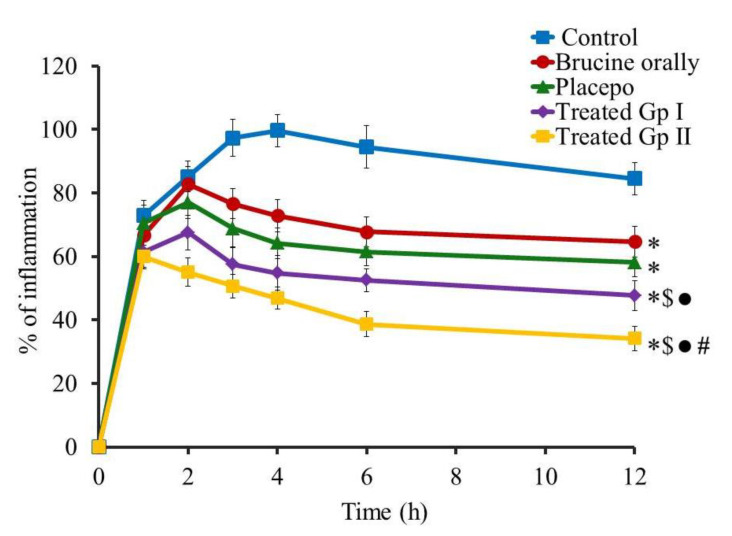
Representing the anti-inflammatory effect of various formulations on rat hind paw edema. Results are expressed as mean with the bar showing SD (n = 6). * *p* < 0.05 versus control-treated group; $ *p* < 0.05 versus Brucine orally treated group; ● *p* < 0.05 versus placebo treated group and # *p* < 0.05 versus treated GP I.

The inspection of anti-inflammatory action on carrageenan-induced rat hind paw treated with formulations encapsulating Brucine was executed, and the profile of the *in vivo* study was displayed in Figure 10. Vigilant observation of the result showed that the maximum percentage of inflammation was reached following 4 h in the control group (99.7 ± 5.1%), which displayed a significant difference when compared to all other groups under examination (*p* < 0.05). In addition, after 12 h of the study, a significantly higher inflammation was still observed between the control group (84.5 ± 5.2%) and other groups in the study (*p* < 0.05). Likewise, maximum % of inflammation was detected following 2 h in groups treated with Brucine orally and the placebo-treated group, and no significant difference was detected between them during the whole experiment (*p* < 0.05). However, both groups exhibited significant reduction\compared to control group 3 h following the initiation of the experiment (*p* < 0.05). This finding confirmed the effect of the placebo-treated group in diminishing the inflammation and suggested the role of jojoba oil in reducing the inflammation. The result is in agreement with Habashy et al., who demonstrated the efficiency of jojoba in lowering inflammation in various examination models [47]. On the other hand, it was noted that % of inflammation reached in the Brucine orally treated group (64.7 ± 4.8%) and placebo-treated group (58.2 ± 4.6%) was significantly higher when compared with treated GP I (47.7 ± 4.8%) and treated GP II (34.2 ± 3.8%) (*p* < 0.05). Additionally, it was important to observe that at 6 and 12 h following the initiation of the experiment, treated GP II showed marked significant reduction of the inflammation, compared to all formulations under investigation (*p* < 0.05), which affirm the role of emulgel and its constituent in improving the anti-inflammatory effect of liposome and supports the synergistic action between Brucine and jojoba oil. This result is similar to that of Ibrahim and Shehata who proved that niosomal emulgel could considerably augment the anti-inflammatory effect of Ketorolac [45].

## 3. Conclusions

In the present exploration, various PEGylated Brucine liposomal formulations were developed using thin film hydration method. The quality by design approach contributes to optimizing the formulation to be combined into jojoba-oil-based emulgel to form PEGylated Brucine liposomal emulgel. The formulation revealed respectable physical characterization, improved skin permeation performance and significant anti-inflammatory action. The result elucidated the incredible effect of jojoba oil toward the *in vivo* behavior of liposomal formulation that suggests the synergistic effect between Brucine and jojoba oil. Ultimately, liposomal emulgel could be considered as a prospective nanocarrier that successfully delivers the drug topically. 

## 4. Materials and Methods

### 4.1. Material

Brucine was obtained from Alpha Chemika (Mumbai, India). Egg phosphatidyl choline (EPC) and cholesterol were purchased from Sigma Aldrich (St. Louis, MO, USA). Poly ethylene glycol-distearoylphosphatidyl ethanolamine (DSPE-PEG 2000) was procured from Lipoid LLC (Newark, NJ, USA). Jojoba oil was obtained from NOW^®^ Essential Oils (NOW Foods, Bloomingdale, IL, USA). Ethanol, chloroform, polysorbate 80 (Tween 80) and Sodium carboxy methylcellulose (Na CMC) were acquired from Sigma-Aldrich Co. (St Louis, MO, USA). All other reagents were of the finest grade available.

### 4.2. Experimental Design

Optimizing the fabricated PEGylated Brucine loaded liposome was carried out using Central Composite Design (CCD), which is one tool of Response Surface Methodology (RSM). Fundamentally, two-factor, two-level (2^2^) factorial design was constructed using two independent factors, EPC concentration (A) and cholesterol concentration (B), in which two levels were selected, low (−1) and high (+1), as illustrated in Table 5. The action of these independent variables on the investigated dependent variables was evaluated by operating Design-Expert software version 12.0 (Stat-Ease, Minneapolis, MN, USA). The examined dependent variables were particle size (R_1_), encapsulation efficiency EE (R_2_) and drug *in vitro* release after 6 h (R_3_). The obtained data were analyzed via an analysis of variance (ANOVA) test followed by assembling model graphs and actual mathematical equations that clarify the influence of the independent variables on the explored dependent variables.

### 4.3. Preparation of PEGylated Brucine-Loaded Liposome

Thin film hydration method that was previously described by Knudsen et al. was executed in order to develop different liposomal formulations [48]. Fifty milligrams of Brucine, ten milligrams of DSPE-PEG 2000 and a specified amount of EPC and cholesterol, as mentioned in Table 1, was added to a round bottom flask and mixed with a 6 mL ethanol: chloroform mixture (1:2). The flask was attached to a rotary evaporator (Heidolph, GmbH, Co. KG, Schwabach, Germany) for 2 h at 100 rpm maintained at 60 °C and operated till complete evaporation of the solvent and formation of thin lipid film on the inner wall of the round-bottom flask. The obtained lipid film was rehydrated with 5 mL phosphate buffer pH 7.4 for 30 min while vortexing using a classic advanced vortex mixer (VELP Scintifica, Usmate Velate, Italy). Next, the resultant dispersion was subjected to sonication for 30 s utilizing probe sonicator (XL-2000, Qsonica, Newtown, CT, USA) to obtain proper particle size. Eleven experimental formulations were fabricated using (CCD) alongside the values of their observed response as clarified in Table 1.

### 4.4. Characterization of PEGylated Brucine Loaded Liposome

#### 4.4.1. Determination of Particle Size

Particle size of the fabricated liposomes was analyzed using Zetasizer apparatus (Malvern Instruments Ltd., Worcestershire, UK). Dynamic light scattering was used for evaluating the formulations keeping a scattering angle (90°) and temperature 25 °C [49].

#### 4.4.2. Encapsulation Efficiency (EE)

A centrifugation method using centrifuge (Andreas Hettich GmbH, Co. KG, Tuttlingen, Germany) was applied to estimate the percentage of Brucine encapsulated into the PEGylated liposome. A sample of the preparation was added into Amicon^®^ ultra-4 (Ultracel-10K, Merk Millipore Ltd., County Cork, Ireland) and allowed to be centrifuged at 6000 rpm maintained at 4 °C for 1 h. The filtrate was collected, diluted and measured spectrophotometrically at λ_max_ 262 nm using spectrophotometer (UV Spectrophotometer, JENWAY 6305, Bibby Scientific Ltd., Staffordshire, UK) for detecting the free drug [50]. EE% was calculated as follows:

% EE = ((Total − Free)/Total) × 100

### 4.5. In Vitro Drug Release from Different Liposomal Preparations

This investigation was designed to identify the percentage of Brucine released from the prepared PEGylated liposomal formulations. In this context, the ERWEKA dissolution system (ERWEKA, GmbH, Heusenstamm, Germany) functioned in order to perform the experiment. Briefly, liposomal samples were kept in glass tubes that closed from one side with a (Dialysis membrane Spctra/por^®^ (MWCO 2000–15,000), New Brunswick, NJ, USA). The tubes were attached to the apparatus and immersed into the release media, which formed of phosphate buffer pH 7.4 maintained at 37 ± 0.5 °C and attuned at rotation speed 50 rpm. Two milliliters of the samples were taken at certain time intervals (0.25, 0.5, 1, 2, 4 and 6 h) and checked spectrophotometrically for the absorbance at λ_max_ 262 nm. The checked samples were substituted with the same volume of the vehicle [51].

### 4.6. Stability Studies of Optimized Liposomal Formulation

Stability of the optimized PEGylated Brucine liposomal formulation was certified relative to various parameters including the particle size, EE and *in vitro* drug release. The investigation was executed in agreement with the guiding principle of International Conference on Harmonization (ICH). The sample was stored at two environments, 4 ± 1 °C and at 25 ± 1 °C for 1 and 3 months.

### 4.7. Preparation of PEGylated Liposomal Emulgel Encapsulating Brucine

**Figure 11:**
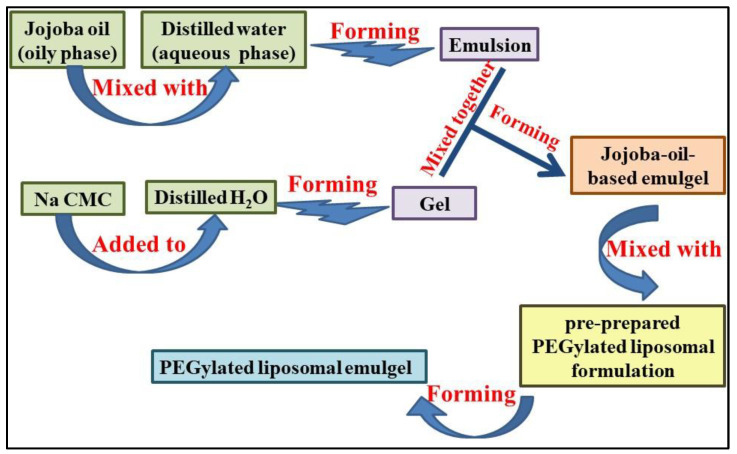
A schematic representation demonstrating the steps of manufacturing PEGylated liposomal emulgel incorporating Brucine.

As mentioned previously, topical preparation should exhibit proper viscosity in order to spread properly and not be detached easily from the affected area of the skin. For that reason, the optimized liposomal formulation was integrated into a pre-prepared jojoba-oil-based emulgel. In short, 0.5 g Na CMC was added to 10 mL distilled water and allowed to stir till homogenous gel was attained. On the other side, emulsion was prepared using 1 g jojoba oil that mixed for 5 min with 1 g Tween 80. Afterward, aqueous phase was gently added over the oily phase with constant vortexing using classic advanced vortex mixer (VELP Scientifica, Usmate Velate, Italy) for 10 min till white emulsion was obtained. To attain the desired emulgel, the formulated emulsion was added to the pre-formulated gel and mixed with support of a mixer (Heidolph RZR1, Heidolph Instruments, Schwabach, Germany) till the development of homogenous jojoba-oil-based emulgel [52]. For developing PEGylated liposomal emulgel encapsulating Brucine, the pre-prepared liposomal formulation was mixed with the developed emulgel using a mixer (Heidolph RZR1, Heidolph Instruments, Schwabach, Germany) till the desired formulation was obtained. Figure 11 displayed an illustrative scheme for the method of developing PEGylated liposomal emulgel loaded with Brucine.

### 4.8. Estimating the Features of Developed PEGylated Liposomal Emulgel Loaded with Brucine

#### 4.8.1. Physical Inspection

This inspection was performed via visual assessment of the color and homogeneity that related to the developed PEGylated liposomal emulgel loaded with Brucine.

#### 4.8.2. Validation of pH Value

The pH value of the topical formulation is very critical parameter to ensure the safety of the preparation. This evaluation was carried out using Standardized pH meter (MW802, Milwaukee Instruments, Szeged, Hungary) [53].

#### 4.8.3. Spreadability Test

The current experiment is important for checking the competence of the formulation to spread evenly when smeared on the affected area of the skin. This is practically accomplished by measuring the spreading diameter where 1 g of the liposomal emulgel was added to be in between two glass slides (25 cm × 25 cm). A definite load of about (500 g) was put over the slides for 1 min. The spreading diameter of the formulation was assessed to provide an indication about the spreadability [54].

#### 4.8.4. Viscosity

The measurement of the formulation viscosity was performed in order to determine its rheological behavior. This evaluation was employed using Brookfield viscometer (DV-II+ Pro., Middleboro, MA, USA) utilizing spindle R5 that rotate at 0.5 rpm at 25° [55].

### 4.9. In Vitro Drug Release from Liposomal Emulgel

The technique previously stated in Section 4.5 was followed in order to validate the percentage of Brucine released from the developed PEGylated liposomal emulgel formulation and compared to the free drug and the liposomal preparation [51].

### 4.10. Animal

All animal experiments were carried out using male Wistar rats of about 200–220 g. Housing condition of the animals was controlled to be switched every 12 h between light and dark cycles, and the temperature was kept at 25 ± 2 °C. The animals were obtained from the animal breeding center at the College of Science, King Faisal University. All experiments were conducted in agreement with the regulations and recommendations of Research Ethics Committee (REC) of Ha’il University (20455/5/42).

### 4.11. Ex Vivo Study

#### 4.11.1. Animal Skin Preparation

Animal skin required for present study was obtained from male Wistar rats. The hair at the dorsal part of the animal was shaved carefully using an electric clipper followed by scarifying the rats and separating the skin. The adipose tissue was removed from the skin then it was preserved at 4 °C in phosphate buffer (pH 7.4) [45].

#### 4.11.2. *Ex Vivo* Permeation Study

Permeation capability of the drug from Brucine suspension, optimized liposome and liposomal emulgel through animal skin was determined using amended Franz diffusion cells organized in our lab and used previously [46,56]. One hundred milliliters of phosphate buffer pH 7.4 containing 0.02% sodium azide was used, representing the release media and kept at 37 ± 0.5 °C. The rat skin was well attached to a glass tube that was held in the apparatus and suspended into the vehicle. The skin was used instead of cellophane membrane, where the upside stratum corneum was adhered to the sample, whereas the skin dermis was facing the media. All cells were shielded with Parafilm (Bemis, Oshkosh, WI, USA) in order to avoid evaporation of media. The system was operated and allowed to rotate constantly at 100 rpm [57]. Aliquots were taken to be analyzed spectrophotometrically at 0.25, 0.5, 0.75, 1, 2, 3, 4, 5 and 6 h. Certain parameters associated with the permeation across the skin were calculated including steady state transdermal flux (SSTF) and enhancement ratio (ER). SSTF characterizes the amount of permeated drug/(area × time); however, ER designates SSTF of test/SSTF of control. The experiment was assessed in triplicates.

### 4.12. In Vivo Study

#### 4.12.1. *In Vivo* Skin Irritation Test

It is very important that topical preparations be safe and that no skin sensitivity is shown. To verify this, a skin irritation test was done using male Wistar rats that were prepared one day before beginning the study. The examined liposomal emulgel formulation was applied gently over the skin after being shaved using an electric clipper. Animals were kept under observation for 7 days to distinguish any sensitivity response including inflammation, irritation, erythema or edema. The detected response was clarified on the basis of a scale that ranged from 0, 1, 2 and 3 where it represents no reaction, minor reaction, moderate reaction or severe erythema that might be accompanied with edema, respectively [42].

#### 4.12.2. *In Vivo* Anti-Inflammatory Study: Carrageenan-Induced Rat Hind Paw Edema Method

The anti-inflammatory influence of Brucine encapsulated into liposomal emulgel was appraised using male Wistar rats that went through a carrageenan-induced rat hind paw edema protocol as conducted previously by Shehata et al. [58]. Edema was initiated into the rat hind paw 30 min prior to the commencement of the study using subcutaneous injection of 0.5% *w/v* carrageenan in saline into the left hind paw [59]. Rats were arbitrarily categorized into 5 groups; each group carrying 6 animals as follows: 

Group I was related to the control group, which is incited with inflammation only without treatment. 

Group II treated orally with Brucine suspension (10 mg/kg) [60]. 

Group III was placebo that treated with PEGylated liposomal emulgel with no drug. 

Group IV was treated with jojoba oil emulgel (Treated GP I). 

Group V was treated with PEGylated liposomal emulgel formulation (Treated GP II). 

The inflammatory response was assessed at diverse time (0, 1, 2, 3, 4, 6 and 12 h). Meanwhile, the distinctions in the thickness of rat hind paw following topical application of the examined formulations were measured by means of digital caliber. The % of inflammation was calculated from the following equation [61]:

% of inflammation = ((Th_t_ − Th_0_)/Th_0_) × 100

where Th_t_ designates the thickness of carrageenan treated hind paw however Th_0_ describes the hind paw at time zero.

### 4.13. Statistics

All investigations were performed at least three independent times, and the results were accompanied with mean ± SD. To distinguish the statistical differences between the groups, a Student’s *t*-test was done. A one-way analysis of variance (ANOVA) followed by the least significant difference (LSD) as a post hoc test was employed to compare data and state the statistical significance. These assessments were designed using SPSS statistics software, version 9 (IBM Corporation, Armonk, NY, USA). If *p*-value < 0.05, it is considered to be statistically significant.

## Figures and Tables

**Table 1:** The independent variables used for optimizing different PEGylated liposomal formulations and the detected dependent variables.

Formulation	Independent Variables		Dependent Variables		
	A (mg)	B (mg)	R_1_ (nm)	R_2_ (%)	R_3_ (%)
F1	75	17.07	248 ± 2.0	51.4 ± 3.8	61.2 ± 4.9
F2	100	15	271 ± 5.3	65.8 ± 3.9	59.2 ± 4.1
F3	50	15	207 ± 1.9	45.8 ± 1.6	72.5 ± 3.7
F4	110.35	10	287 ± 4.5	68.3 ± 3.5	56.3 ± 3.9
F5	75	10	229 ± 3.2	54.2 ± 1.9	64.3 ± 5.1
F6	50	5	202 ± 3.5	49.3 ± 3.0	75.4 ± 2.6
F7	75	10	227 ± 2.9	55.0 ± 1.3	65.0 ± 4.6
F8	75	2.92	210 ± 2.7	60.0 ± 2.5	71.2 ±4.9
F9	39.64	10	191 ± 2.9	41.6 ± 2.1	79.2 ± 5.5
F10	75	10	223 ± 3.2	56.2 ± 2.3	67.1 ± 4.5
F11	100	5	262 ± 3.6	71.6 ± 1.6	62.8 ± 4.4

A: EPC concentration; B: cholesterol concentration; R_1_: particle size; R_2_: EE%; R_3_: % drug *in vitro* release.

**Table 2:** Results of statistical analysis of all dependent variables R_1_, R_2_ and R_3_.

Source	R_1_		R_2_		R_3_	
	F-Value	*p*-Value	F-Value	*p*-Value	F-Value	*p*-Value
Model	72.58	<0.0001 *	119.61	<0.0001 *	71.93	<0.0001 *
A-EPC	135.92	<0.0001 *	223.17	<0.0001 *	127.83	<0.0001 *
B-Cholesterol	9.24	<0.0161 *	16.04	0.0039 *	16.03	0.0039
Lack of Fit	8.53	0.1086	4.39	0.1971	1.75	0.4069
R^2^ analysis						
R²	0.9478		0.9676		0.9899	
Adjusted R²	0.9347		0.9595		0.9797	
Predicted R²	0.8948		0.9329		0.9341	
Adequate Precision	22.3246		28.6059		29.1829	

A, EPC concentration (mg); B, cholesterol concentration (mg); R_1_, particle size (nm); R_2_, EE (%); R_3_, *in vitro* release (%); * significant.

**Table 3:** Predicted and observed results of the optimized PEGylated Brucine liposomal formulation.

Independent Variables	Symbol	Goal
EPC	A	In range
Cholesterol	B	In range
Dependent variables	Predicted results	Observed results
R_1_ (nm)	224.8 ± 7.8	221.8 ± 3.01
R_2_ (%)	59.22 ± 1.89	57.66 ± 3.06
R_3_ (%)	69.14 ± 1.82	67.96 ± 2.65

**Table 4:** Skin permeation parameters of different developed formulations.

Formula	SSTF µg/cm^2^·h	ER
Free Brucine	0.202 ± 0.015	1
PEGylated liposome	0.321 ± 0.028 * #	1.603 ± 0.142 * #
Liposomal emulgel	0.47 ± 0.035 *	2.33 ± 0.174 *

Values are expressed as mean ± SD. * *p* < 0.05 compared to free Brucine suspension, # *p* < 0.05 compared to PEGylated liposomal emulgel formulation.

**Table 5:** CCD data showing independent variables and their level of variation.

Independent Variable	Character	Level of Variation	
		−1	+1
EPC concentration (mg)	A	50	100
Cholesterol concentration (mg)	B	5	15

## Data Availability

Not applicable.
